# Transmission of ciprofloxacin resistance in *Salmonella* mediated by a novel type of conjugative helper plasmids

**DOI:** 10.1080/22221751.2019.1626197

**Published:** 2019-06-06

**Authors:** Kaichao Chen, Ning Dong, Edward Wai-Chi Chan, Sheng Chen

**Affiliations:** a Shenzhen Key lab for Food Biological Safety Control, Food Safety and Technology Research Center, Hong Kong PolyU Shen Zhen Research Institute, Shenzhen, People’s Republic of China; b State Key Lab of Chirosciences, Department of Applied Biology and Chemical Technology, The Hong Kong Polytechnic University, Kowloon, Hong Kong

**Keywords:** *Salmonella*, ciprofloxacin resistance, PMQR genes, conjugative helper plasmid, plasmid integration

## Abstract

Ciprofloxacin resistance in *Salmonella* has been increasingly reported due to the emergence and dissemination of multiple Plasmid-Mediated Quinolone Resistance (PMQR) determinants, which are mainly located in non-conjugative plasmids or chromosome. In this study, we aimed to depict the molecular mechanisms underlying the rare phenomenon of horizontal transfer of ciprofloxacin resistance phenotype in *Salmonella* by conjugation experiments, S1-PFGE and complete plasmid sequencing. Two types of non-conjugative plasmids, namely an IncX1 type carrying a *qnrS1* gene, and an IncH1 plasmid carrying the *oqxAB*-*qnrS* gene, both ciprofloxacin resistance determinants in *Salmonella*, were recovered from two *Salmonella* strains. Importantly, these non-conjugative plasmids could be fused with a novel Incl1 type conjugative helper plasmid, which could target insertion sequence (IS) elements located in the non-conjugative, ciprofloxacin-resistance-encoding plasmid through replicative transcription, eventually forming a hybrid conjugative plasmid transmissible among members of *Enterobacteriaceae*. Since our data showed that such conjugative helper plasmids are commonly detectable among clinical *Salmonella* strains, particularly *S.* Typhimurium, fusion events leading to generation and enhanced dissemination of conjugative ciprofloxacin resistance-encoding plasmids in *Salmonella* are expected to result in a sharp increase in the incidence of resistance to fluoroquinolone, the key choice for treating life-threatening *Salmonella* infections, thereby posing a serious public health threat.

## Introduction

*Salmonella* is a leading cause of food-borne illness worldwide [1]. Intestinal salmonellosis is usually self-limiting and resolves within a week, while in patients at the extremes of age and those who are immunocompromise, it can cause systematic infections leading to death. Antimicrobial therapy (e.g. ciprofloxacin in adults and ceftriaxone in children) can be lifesaving in these patients [2]. In recent years, the increased usage of fluoroquinolone in livestock for control and treatment of infectious diseases have caused a dramatic increase in the incidence of ciprofloxacin resistance in *Salmonella* in both clinical and food isolates in various countries in recent years, particularly in China and the adjacent areas [3].

Ciprofloxacin resistance is mainly attributed to double mutations in the *gyrA* gene and single mutation in the *parC* gene in *Salmonella* [4,5]. Efflux pumps and the presence of plasmid-mediated quinolone resistance (PMQR) determinants including *qnr*, *aac(6′)-Ib-cr*, *oqxAB* and *qepA* genes have also been regarded as contributive factors of development of low-level resistance to nalidixic acid in *Salmonella* [6–11]. The *oqxAB* and *aac(6′)-Ib-cr* genes often co-exist in the same strain, and may be associated with an increase in incidence of ciprofloxacin resistance in clinical *Salmonella* strains in recent years [12]. A large proportion of ciprofloxacin-resistant *Salmonella* currently harbour only a single mutation or even no mutation in the *gyrA* gene or other target genes, but instead often carry one or more PMQR genes [13]. Furthermore, some *Salmonella* isolates have been shown to carry multiple PMQR genes in their chromosome or in non-conjugative plasmids, resulting in progressive development of ciprofloxacin resistance. Recently, two types of conjugative ciprofloxacin-resistance-encoding plasmids have been reported in *Salmonella* [14]. In addition, a conjugative fusion plasmid that carried the *bla*_CTX-M_ and PMQR genes, encoding resistance to both ceftriaxone and ciprofloxacin respectively, was reported. This fusion plasmid was found to exist in a dynamic form in *Salmonella* in such a way that the strain contained both the fusion plasmid and two daughter plasmids [15]. In this study, we report the emergence of a type of broad-host range conjugative helper plasmid that could be fused with a non-conjugative plasmid carrying multiple PMQR genes, thereby transforming the latter into a conjugative, ciprofloxacin resistance-encoding plasmid. These data indicated that ciprofloxacin resistance in *Salmonella* mediated by many new mechanisms has evolved quickly and continuous monitoring is urgently needed.

## Materials and methods

### *Salmonella* isolation

*Salmonella* were isolated from retail meat products purchased from supermarkets and wet markets in Shenzhen, China during the period January 2014 to January 2015 as reported in the previous study [16]. In this study, five *Salmonella* isolates that were able to transfer their ciprofloxacin resistance phenotype to *E. coli* strain J53 via conjugation were further characterized in this study. In addition, a total of 1985 clinical *Salmonella* isolates including various serotypes recovered from patients in the Second Affiliated Hospital of Zhejiang University during the period 2004–2015 were used to screen for presence of the helper plasmid in these clinical *Salmonella* strains.

### Antimicrobial susceptibility testing

Susceptibility to 14 antimicrobials listed in Table 1 was determined for five *Salmonella* strains and their transconjugants (Table 1) using the agar dilution method according to the Clinical and Laboratory Standards Institute (CLSI) guidelines [17]. *Escherichia coli* strain ATCC 25922 was used as a quality control.

**Table 1.** Genetic and phenotypic characteristics of ciprofloxacin-resistant Salmonella strains and the corresponding transconjugants.

Strain ID	Species	PMQR genes	Plasmids (∼kb)	MIC (µg/ml)											
				AMK	CTX	CIP	KAN	OLA	STR	CRO	TET	CHL	NAL	AMP	SXT
25922	*E. coli*	–	–	4	0.06	0.0075	2	16	8	0.03	2	4	1	8	1
J53(Azi^R^)	*E. coli*	–	–	≤0.5	≤0.015	0.015	≤0.5	4	2	≤0.015	0.5	1	2	2	4
Sa48	*S.* London	–	–	1	0.06	0.075	8	16	>128	0.06	>32	64	8	16	8
Sa21	*S.* Agona	*qnrS*	87, 28	2	0.12	1	2	32	128	0.12	>32	4	16	>64	4
TC-Sa21	*E. coli* J53	*qnrS*	115	≤0.5	0.06	0.5	≤0.5	16	64	0.03	16	2	16	>64	4
^#^Sa48-TC-Sa21	*S.* London	*qnrS*	115	≤0.5	0.06	1	≤0.5	16	64	0.03	16	2	16	>64	4
*Sa27	*S.* Derby	*qnrS-oqxAB*	186, 83	1	0.12	4	2	256	>128	0.12	>32	>64	16	>64	32
TC-SA27	*E. coli* J53	*qnrS-oqxAB*	270	≤0.5	0.06	2	≤0.5	128	128	0.03	>32	2	64	>64	16
Sa48-TC-Sa27	*S.* London	*qnrS-oqxAB*	270	≤0.5	0.06	2	≤0.5	128	128	0.03	>32	2	64	>64	16

Notes: AMK, amikacin; CTX, cefotaxime; CIP, ciprofloxacin, KAN, kanamycin; OLA, olaquidox; STR, streptomycin; CRO, ceftriaxone; TET, tetracycline; CHL, chloramphenicol; NAL, nalidixic acid; AMP, ampicillin; MRP, meropenem; SXT, trimethoprim/sulfamethoxazole.

^#^Sa48-TC-Sa21 refers to *Salmonella* conjugatant generated through conjuation of TC-Sa21 to *Salmonella* strain, Sa48, to conjugate the conjugative palsmid back to *Salmonella*.

*Sa26, Sa136, Sa154, which were all S. Derby and shared identical PFGE profile as Sa27 [16], and their transconjugants exhibited similar plasmid profile as Sa27 and TC-Sa27.

### Conjugation experiments

The transmission efficiency of PMQR genes was assessed by performing the conjugation experiment, using the filter mating method as previously described, in which the *E. coli* strain J53(AZIR) was used as a recipient strain [13]. Transconjugants were selected on EMB agar containing sodium azide (100 μg/ml) and either cefotaxime (2 μg/ml) or ciprofloxacin (0.5 μg/ml). To determine whether ciprofloxacin-resistance encoding plasmids, which have been acquired by *E. coli J53* transconjugants, can be further transferred to a *Salmonella* strain, conjugation was also performed by using *E. coli* J53 strains as donor and *S.* London strain Sa48 as recipient strain; ciprofloxacin resistant *Salmonella* transconjugants were selected on XLT4 plates containing ciprofloxacin (0.5 µg/ml) and chloramphenicol (16 µg/ml).

### Plasmid sequencing and bioinformatics analyses

To gain further understanding of the genetic features of these mobile resistance elements, plasmids recovered from both parental strains (Sa21 and Sa27) and their corresponding transconjugants were subjected to complete sequencing using both the Illumina and PacBio RSII single-molecule real-time (SMRT) sequencing platforms. The Illumina paired-end libraries were constructed with the NEBNext Ultra DNA Library Prep Kit for Illumina (NEB) and sequenced on an Illumina NextSeq 500 platform. SMRT sequencing was performed at Wuhan Institute of Biotechnology, China. De novo assemblies of PacBio RSII reads and Illumina reads were performed by the hierarchical genome assembly process (HGAP, Paciﬁc Biosciences) and the CLC Genomics Workbench (CLC bio, Denmark) respectively. Long assembled contigs obtained from PacBio reads were used to align and join the contigs obtained from the Illumina assembly results. The completed plasmid sequence was confirmed by PCR and then annotated with the RAST tool [18] and the NCBI Prokaryotic Genome Annotation Pipeline (PGAP). Each plasmid was sequenced using both Illumina and PacBio platforms and only high-quality data were used for further analysis.

### Screening of specific types of plasmids in clinical *Salmonella* isolates

Based on the sequences of Incl1 plasmid, one set of primers, Incl1F-TCCTGATTTCATCCACGGCAA and Incl1R- AGGGACATTATTTGCCCGCT, was designed and used to screen for the presence of Incl1 type of plasmid in clinical *Salmonella* isolates using PCR assay.

## Results

### Ciprofloxacin resistance mediated by conjugative plasmids in *Salmonella*

**Figure 1.**
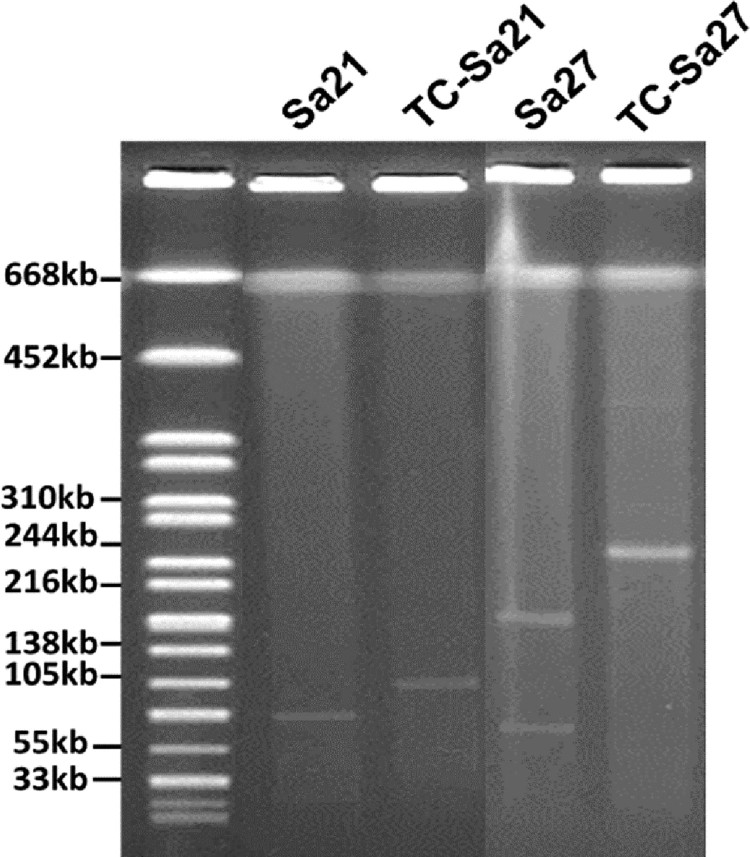
S1-PFGE analysis of plasmid profile of ciprofloxacin-resistant *Salmonella* strains and their corresponding transconjugant*s.* Plamsids in transconjugants were found to be different in size when compared to plasmids in the corresponding parental *Salmonella* strains. In addition, *Salmonella* strains, Sa26, Sa136, Sa154, which were all *S.* Derby and shared a PFGE profile identical to Sa27 [16], and their transconjugants, exhibited a plasmid profile similar to that of Sa27 and TC-Sa27 respectively, therefore they were not shown in the figure.

In our previous study, a total of 157 non-repeated *Salmonella* isolates were recovered from 348 meat samples [16]. Among the 55 Cip^R^*Salmonella* isolates, 14 were able to transfer their ciprofloxacin resistance phenotype to *E. coli* strain J53(AZI^R^). S1-PFGE was performed on these 14 *Salmonella* isolates and their transconjugants. Five out of the 14 *Salmonella* strains were found to carry two plasmids of different sizes, while only one larger size plasmid was detectable in the transconjugants (Figure 1, Table 1). These five Salmonella isolates were then further characterized in this study. Among these five *Salmonella* strains, four belonged to the serotype Derby and shared identical PFGE profile [16] and one was Agona. MIC of ciprofloxacin of the donor strains and the *E. coli* transconjugants was either identical to, or 2–4 folds less than that of the parental strains (Table 1). However, when the conjugative plasmids recovered from *E. coli* J53 transconjugants were transferred back to a ciprofloxacin-susceptible (Cip^S^) *Salmonella* strain, namely *S.* London Sa48, a CIP MIC level, which equalled that of the parent *Salmonella* carrying the test plasmid was recorded, suggesting that these conjugative plasmids could mediate slightly higher MIC in *Salmonella* than in *E. coli* (Table 1). We then tested if temperature could affect the conjugation efficiency of these ciprofloxacin resistance plasmids, with results showing that at 37°C and 42°C, the conjugation efficiency of plasmids in strain Sa21 were 2.4 × 10^−5^ and 1.95 × 10^−5^, respectively; and those of plasmids in strain Sa27 were 7 × 10^−6^ and 2 × 10^−7^, respectively. To gain further understanding of the genetic features of these mobile resistance elements, complete sequences of the plasmids recovered from both parental strains (Sa21 and Sa27) and their transconjugants were obtained.

### Genetic basis of transmission of ciprofloxacin resistance in *Salmonella* strain Sa21

**Figure 2.**
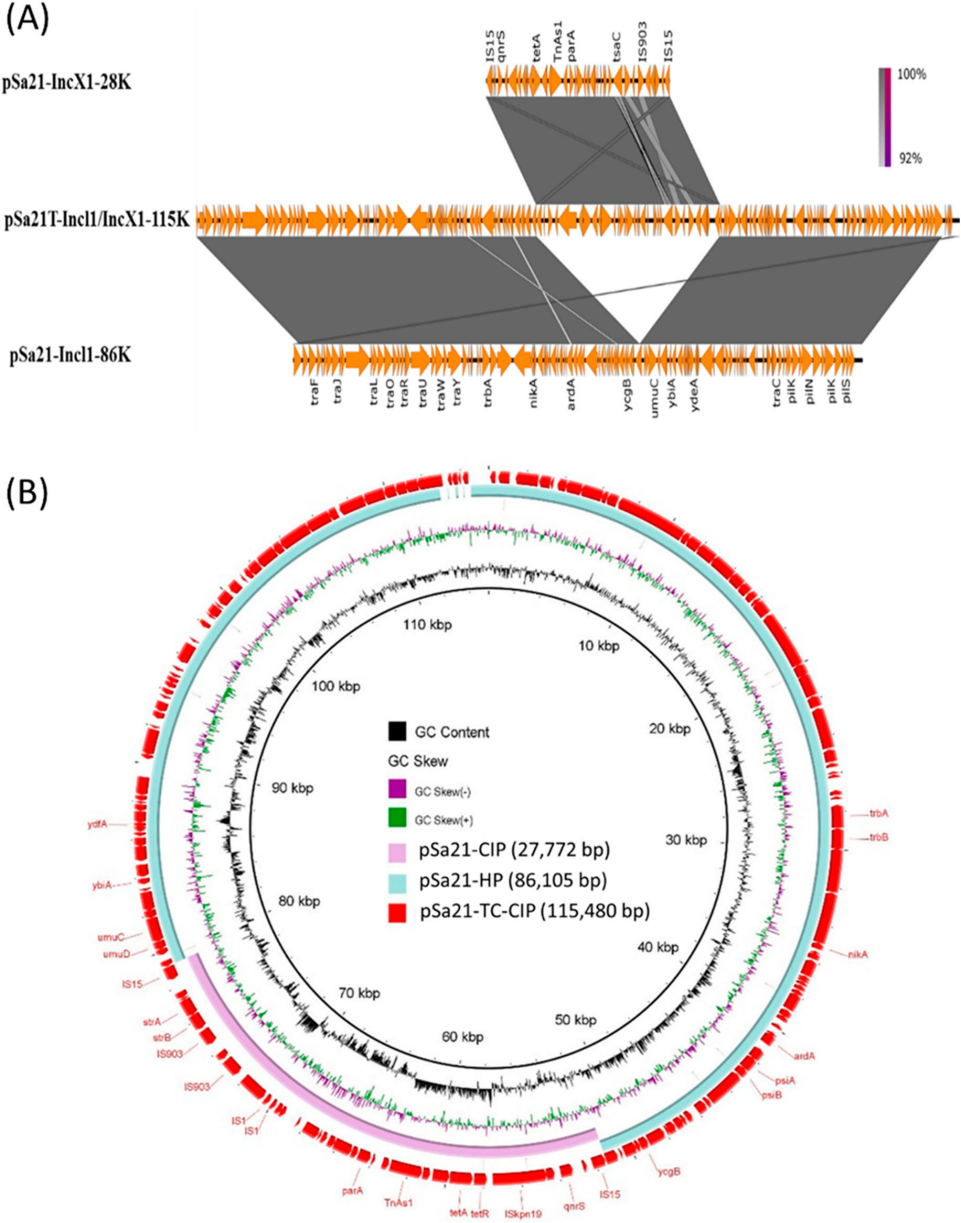
Alignment of plasmids recovered from strain Sa21 using Easyfig (A) and the BLAST Ring Image Generator (BRIG) (B). Plasmid pSa21-TC-CIP was used as a reference in (B); representative genes are labeled. *S.* Agona strain, Sa21, harbours two plasmids, pSa21-HP and pSa21-CIP. The two plasmids were fused during transconjugation and formed a single plasmid pSa21-TC-CIP. The PMQR gene *qnrS* in plasmid pSa21-CIP was incorporated into the new plasmid pSa21-TC-CIP, which became conjugative. Plasmid sequence was generated through combination of both Illumina and PacBio sequencing data.

Among the two plasmids in *S.* Agona strain Sa21, the larger one has a size of 86,105 bp with GC content of 50.2%, and contains 121 CDs. This plasmid was found to belong to the Incl1 plasmid group and carry mainly IncI1 genes for conjugative functions, but not any antibiotic resistance genes (Figure 2). BLAST analysis showed that this type of plasmid had previously been reported in *E. coli* and *Salmonella*, with pCE-R2-11-0435_92 (accession: CP016520), which was isolated from a *S.* Heidelberg strain in chicken meats in Canada, and pEC15I_2 (accession: KU932030), which was isolated from a pig-borne *E. coli* strain Finland, exhibiting the highest degree of sequence homology (Figure 2).

The smaller plasmid was found to be a IncX1 type of plasmid with 27,772 bp in size and a GC content of 51.1%; this plasmid was found to contain 41 CDs including a *qnrS1* gene, which was presumably responsible for causing phenotypic ciprofloxacin resistance (Figure 2). We then designated this plasmid as pSa21-CIP. BLASTN of pSa21-CIP did not identify any plasmid of similar structures. Nevertheless, the structure of the region harbouring mobile resistance elements (∼11 kb), which comprised the *qnrS1*and *tetA* genes, was found to be identical to that of a DNA fragment in a conjugative IncX1 plasmid, pEBG1 (KF738053.1), which was recovered from *E. coli* strain 09/22a in Nigeria. On the other hand, a ∼7 kb plasmid backbone region was also found to be homologous to plasmid A (CP010164.1) in a *E. coli* strain (H2) in China.

Only one conjugative plasmid was detectable in transconjugants. This plasmid was found to be 115,480 bp in size with GC content of 50.3%, and carry 157CDs. Sequence analysis revealed that this plasmid was the fusion product of two plasmids in the parental strain Sa21 (Figure 2). This fusion plasmid was associated with an increase in CIP MIC of *E. coli* J53 from <0.01 to 0.5 µg/ml. Transfer of this plasmid back to the Cip^S^*Salmonella* strain Sa48 resulted in a CIP MIC of 1 µg/ml. This plasmid was designated as pSa21-TC-CIP. The observation that only the fusion plasmid pSa21-TC-CIP is conjugative suggests that the original CIP-resistance-encoding plasmid, pSa21-CIP, is not transmissible and needs the other plasmid to mediate its transfer to *E. coli* strain J53 through conjugation. We therefore designated the plasmid mediating the transmission of pSa21-CIP as conjugative helper plasmid pSa21-HP.

**Figure 3.**
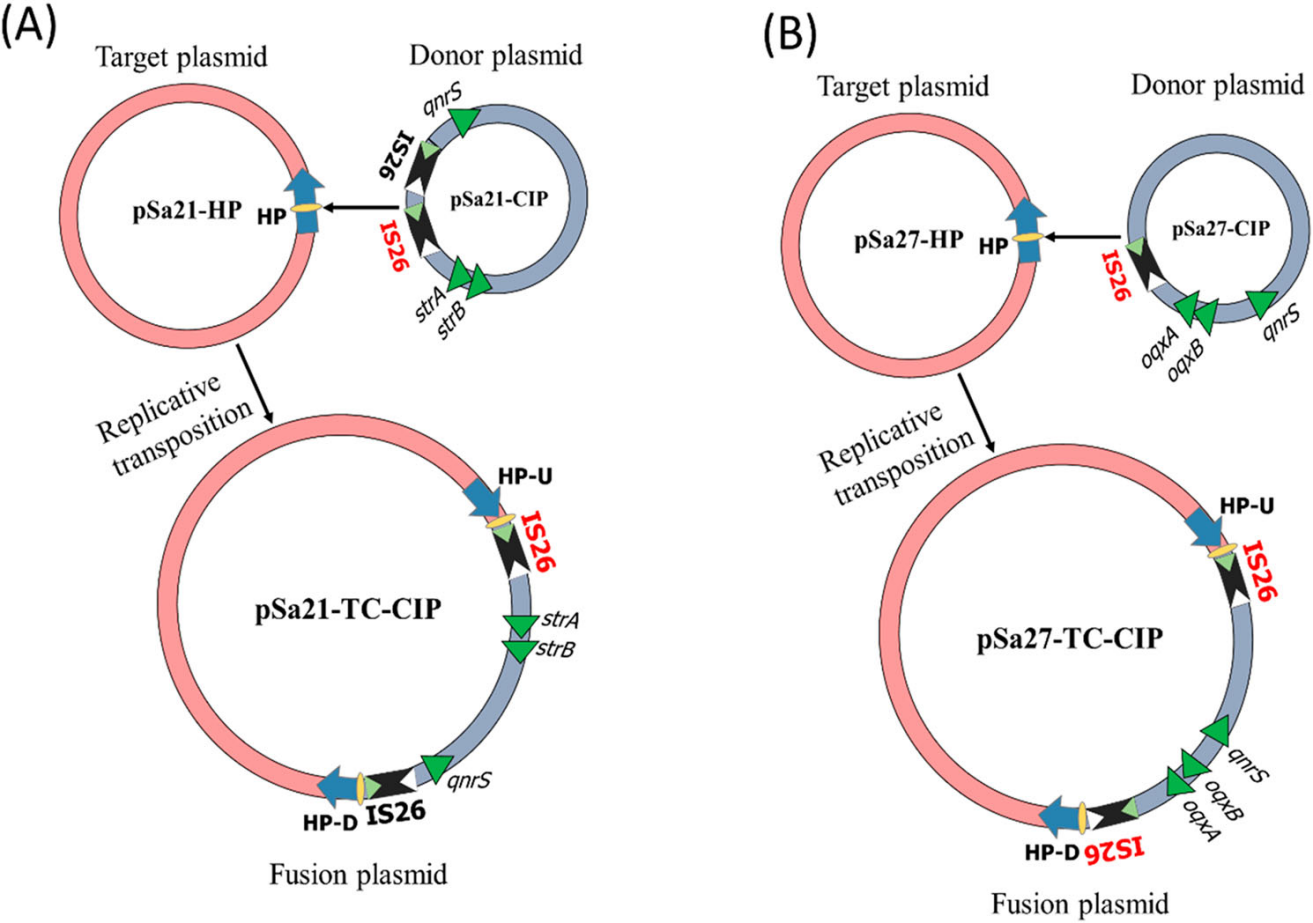
Proposed IS element-mediated plasmid fusion through replicative transposition mechanisms. (A) one copy of IS*26* on pSa27-CIP attacked the hot spot of conjugative helper plasmid, pSa21-HP, leading to formation of a fusion plasmid through intermolecular replicative transposition mechanism; (B) mechanisms of plasmid fusion was similar to that of Sa21, the only difference was that pSa27-CIP contained only one copy of IS*26*.

Detailed sequence analysis of these three plasmids enabled us to predict the possible mechanism of plasmid fusion. First, two IS*26* (Insertion Sequence) elements were found located in pSa21-CIP, one of which might cleave both terminal inverted repeats (TIRs) of the IS*26* elements, resulting in nicks in both strands of DNA and generating 3-Diols groups (3 = –OH) that attack the hot spot of pSa21-HP, leading to formation of a Shapiro intermediate. The DNA complex might then undergo intermolecular replicative transposition in which the target site is duplicated (Figure 3A) [19]. These events result in a 11-bp target site duplication (TSD) (GCAACAGTGCC) in the conjugative helper plasmid.

### Genetic basis of transmission of ciprofloxacin resistance in *Salmonella* strain Sa27

**Figure 4.**
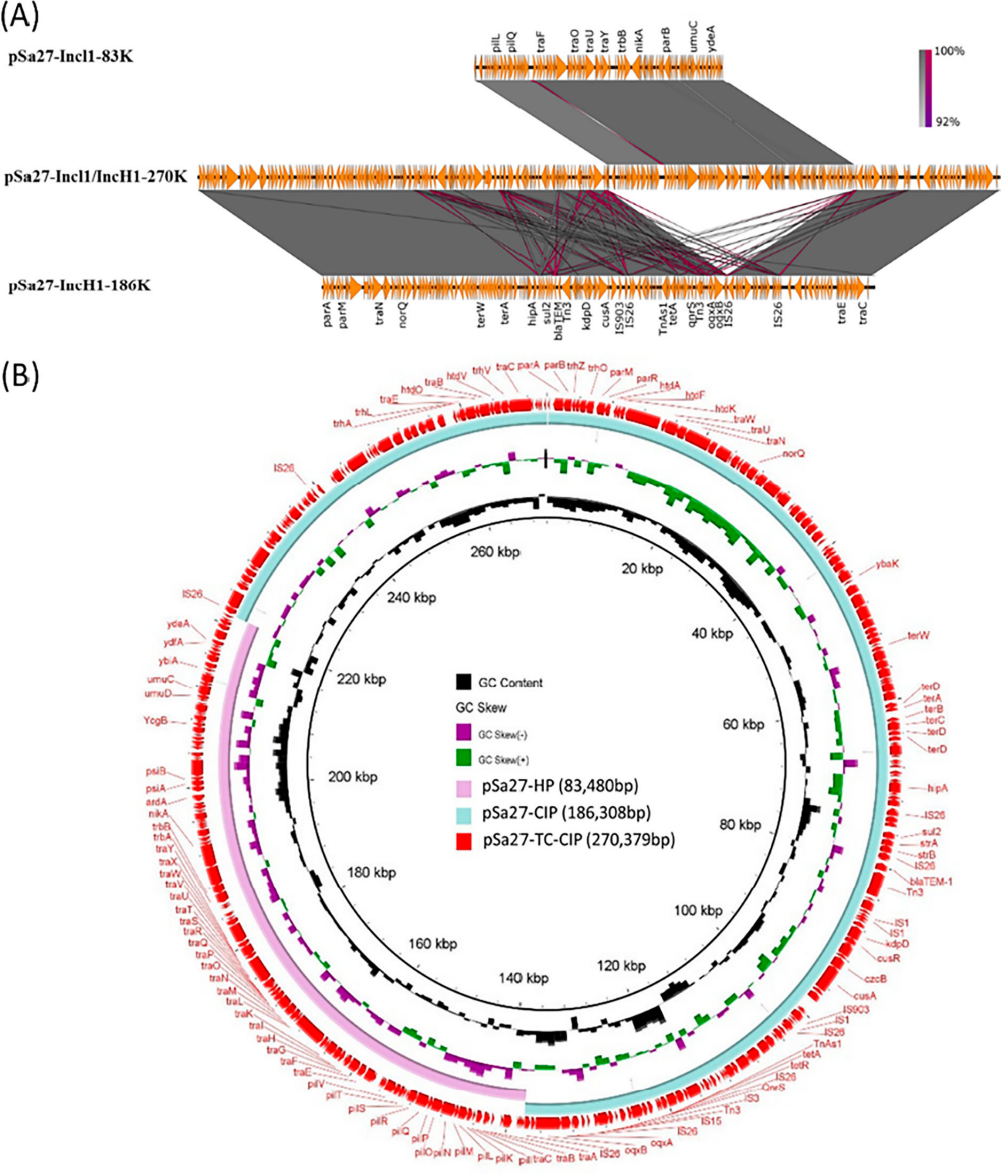
Alignment of plasmids recovered from strain Sa27 using Easyfig (A) and the BLAST Ring Image Generator (BRIG) (B); representative genes are labeled. *S.* Derby strain, Sa27, harboured two plasmids, pSa27-CIP and pSa27-HP. The two plasmids were fused during conjugation process and formed a single plasmid pSa27-TC-CIP. Antimicrobial resistance genes *qnrS*, *bla*_TEM_, *oqxA*, *oqxB*, *sul2* and *tetA* in plasmid pSa27-CIP were integrated to form the new plasmid pSa27-TC-CIP, which became conjugative. Plasmid sequence was generated through combination of both Illumina and PacBio sequencing data.

A similar plasmid fusion event was observed in *S.* Derby strain Sa27. Two plasmids were detected in the parental strain. One plasmid was 186,308 bp in size with a GC content of 46.6%. It carried 226 CDs and belonged to the IncH1 type. This plasmid was shown to harbour two PMQR genes, *oqxAB*-*qnrS*, which might be responsible to ciprofloxacin resistance. We designated this plasmid as pS27-TC-CIP (Figure 4). BLAST analysis of pS27-TC-CIP indicated that it exhibited high-level homology (87% coverage with 99% identity) to the plasmid pECJS-B60-267 (KX254341.1), which was recovered from an animal *E. coli* strain in China, and harboured the *mcr-1*, *fosA3* and *bla*_CTX-M-65_ genes. Compared to pECJS-B60-267, pSa27-TC-CIP was found to have retained the major backbone region of the plasmid but have lost part of the *tra* gene region responsible for plasmid conjugation, as well as the MDR region harbouring the *mcr-1*, *fosA3* and *bla*_CTX-M-65_ genes, yet the plasmid has acquired mobile elements carrying the *oqxAB* and *qnrS* genes.

**Figure 5.**
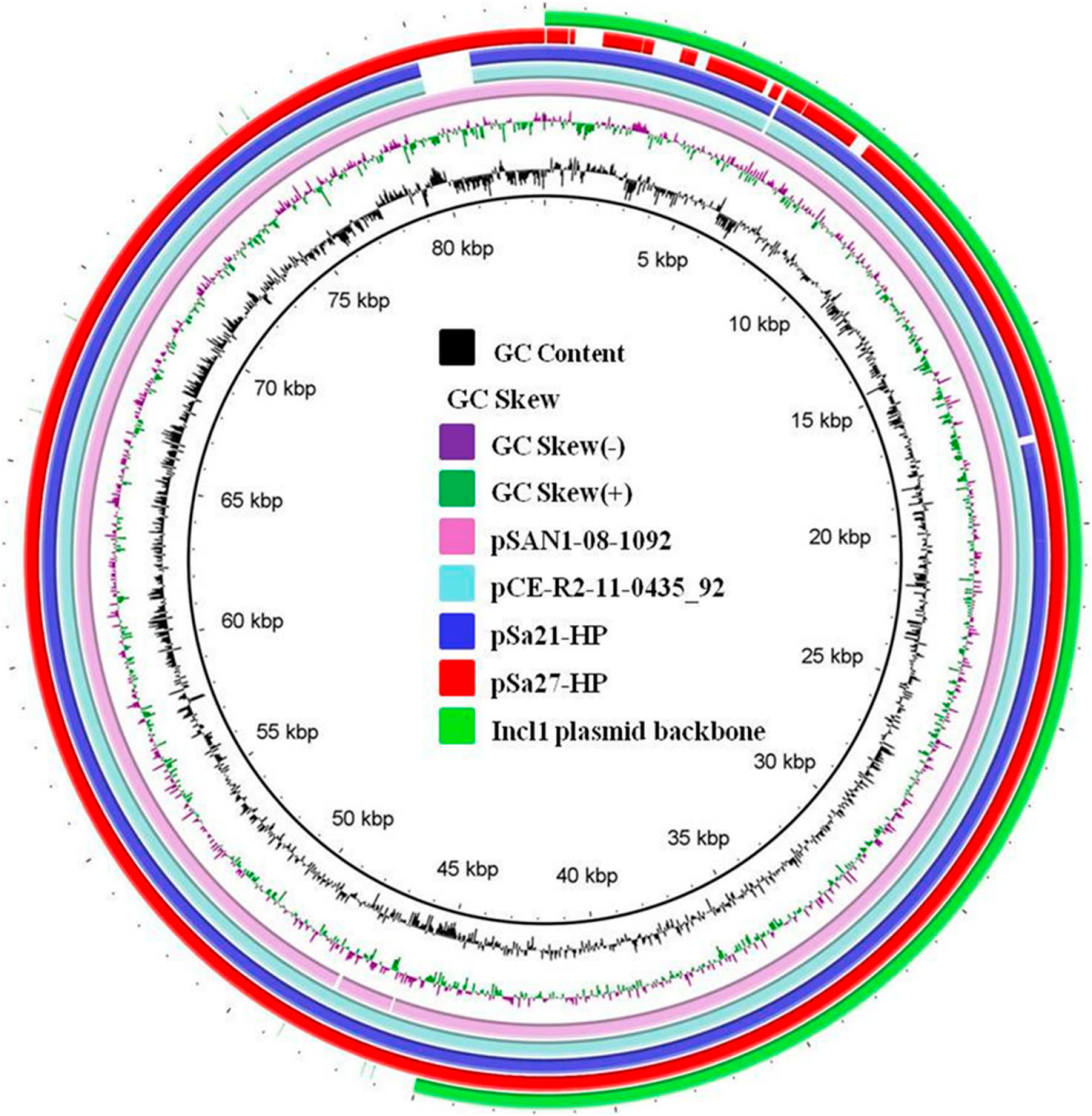
Alignment of Incl1-type conjugative helper plasmids recovered from *Salmonella* using BLAST Ring Image Generator (BRIG). Plasmids pSa21-HP (86,105 bp), pSa27- HP (83,480 bp), pSa44-HP (91,411 bp) of this study and plasmids pCE-R2-11-0435_92 (accession: CP016520), pSal18934a (accession: JF274993) and pEC15I_2 (accession: KU932030) in the NCBI database were analyzed. Sequence of pSa44-HP is used as a reference (red circle) and key genetic loci in this plasmid are labeled. The outmost green circle represents the core structure of IncI1 plasmid. Plasmid sequence was generated through the combination of both Illumina and PacBio sequencing data.

The other plasmid was 83,480 bp in size, with a GC content of 50.3% and carried 114 CDs. It also belonged to Incl1 and was structurally similar to conjugative helper plasmid pSa21-HP, and did not contain any antimicrobial resistance genes (Figure 5). The plasmid in the transconjugant TC-Sa27 was shown to be 270,379 bp in size and designated as pS27-TC-CIP. Sequence alignment of these three plasmids revealed that pSa27-TC-CIP was obtained through fusion between pSa27-HP and pSa27-CIP (Figure 4). Transfer of pS27-TC-CIP to *E. coli* J53 resulted in an increase in CIP MIC from <0.01 to 2 µg/ml, suggesting that pS27-TC-CIP could mediate expression of phenotypic ciprofloxacin resistance in *Salmonella*. Identical plasmid profiles and conjugation events were also observed in the other three *S.* Derby strains, namely Sa26, Sa136 and Sa154, which shared identical PFGE profile (data not shown).

The mechanism of plasmid fusion observed among the three plasmids in strain Sa27 was similar to that of strain Sa21, with the exception that only one copy of IS*26* was detectable in pSa27-CIP. In this case, the IS*26* element might cleave both terminal inverted repeats (TIRs) and attack the hot spot of pSa27-HP, leading to the formation of a Shapiro intermediate. The DNA complex then underwent intermolecular replicative transposition with duplication of the target site (Figure 3B) [19], resulting in an 8-bp target site duplication (TSD) (CTCGCCAG) and acquisition of an additional copy of IS*26* in the conjugative helper plasmid.

### Prevalence of Incl1 conjugative helper plasmids in clinical *Salmonella* strains

PCR assays targeting conservative region of Incl1 conjugative helper plasmid were used to test the presence of the conjugative helper plasmids in clinical *Salmonella* isolates. Incl1 type of plasmids were detected in 32 (20%) out of the 159 Cip^R^*Salmonella* isolates collected in 2015, but not in 1826 clinical *Salmonella* isolates collected during the period of 2004–2014. Among the 32 *Salmonella* isolates carrying the Incl1 conjugative helper plasmid, 28 were *S.* Typhimurium and 4 were *S.* Thompson. Out of these 32 *Salmonella* isolates, 16 were resistant to ciprofloxacin. Conjugation experiments were performed on these 16 *Salmonella* isolates, with results showing that six of them could successfully transfer their ciprofloxacin resistance phenotype to *E. coli* J53, suggesting that this event might be mediated by the helper plasmid. Further study is required to confirm this theory.

## Discussion

Bacterial resistance to fluoroquinolones such as ciprofloxacin is known to be mediated by target gene mutations. Ciprofloxacin resistance in *Salmonella* has previously been attributed exclusively to double *gyrA* mutations and a single *parC* mutation [20]. Due to the low rate of occurrence of *gyrA* double mutations, ciprofloxacin resistance in *Salmonella* remained rare. In 2005, other mechanisms of quinolone resistance such as those due to acquisition of PMQR genes was first reported in *Salmonella*, while these PMQR genes could only mediated quinolone resistance but not resistance to fluoroquinolone such as ciprofloxacin [21]. In recent years, incidence of ciprofloxacin resistance in *Salmonella* increased sharply in certain part of the world such as in China, reaching 30–40% for certain serotypes [11,22]. These newly emerged ciprofloxacin-resistant *Salmonella* strains exhibit CIP MIC between 1 and 16 µg/ml, and often harbour a single *gyrA* mutation, along with PMQR genes. Alternatively, some of such strains harbour multiple PMQR genes without any target gene mutations. The resistance phenotype of such strains are therefore presumably due to the synergistic effects of target mutations and a PMQR gene, or that of multiple PMQR elements that act on fluoroquinolones via the mechanisms of enzymatic inactivation, drug efflux, and competitive inhibition of drug binding, producing a high-level quinolone resistance phenotype in the absence of target gene mutations. To date, PMQR genes in *Salmonella* have consistently been found to be located in either chromosome or non-conjugative plasmids, suggesting that the chance of horizontal transfer of such resistance elements is low.

Non-conjugative resistance-encoding plasmids that carried PMQR genes were observed in this study. These PMQR genes were all flanked by different IS elements including IS*Kpn19*-*qnrS*-IS*15* in the plasmid pSa21-CIP, IS*26*-*oqxAB*-IS*26* and IS*26*-*qnrS*-IS*3* in plasmid pSa27-CIP. A common feature observable among these mobile resistance elements is that they are mostly inserted into the MDR region, often forming circular intermediates that can mediate transposition of the element from one plasmid to another [23]. Upon accumulation in plasmids, these mobile elements, especially those incorporating multiple PMQR genes, confer the ability of the plasmids concerned to encode ciprofloxacin-resistance. Although all ciprofloxacin resistance encoding plasmids were shown to be non-conjugative in this work, we revealed the existence of a novel conjugative helper plasmid that could be fused with the non-conjugative ciprofloxacin resistance-encoding plasmid, rendering it able to undergo conjugation. The resulting hybrid plasmids were proven to be transferrable from *Salmonella* to *E. coli* and vice versa, without affecting the resistance phenotype. Our study also showed that formation of fusion plasmids leading to subsequent transmission to other bacterial strains only happened at 37°C and 42°C, suggesting that these plasmid fusion and transmission events can occur in human and animals.

The conjugative helper plasmids harboured mainly IncI1 genes for conjugative functions, but not antibiotic resistance gene or IS elements. A common feature of these plasmids is that they all contained a hot spot in their sequence recognizable by IS*26* as well as other different types of IS elements, thereby enabling replicative transposition to occur, eventually resulting in fusion with other plasmids. The fusion plasmid would then become conjugative, and hence transferrable from *Salmonella* to *E. coli* and vice versa, as well as among various species of *Enterobacteriaceae*. The phenomenon of plasmid fusion has been reported previously, in which fusion of IncFIB and IncHI2 plasmids in *Salmonella* strains enabled the resulting fusion product to be transferrable to *E. coli* recipient strains. Recently, a plasmid structurally similar to the helper plasmids identified in this study has been reported to be able to form fusion plasmid in *Salmonella*. Such plasmid was shown to be genetically similar to the helper plasmid, but differs by carrying an additional mobile element containing *bla*_CTX-M-130,_ implying that (1) this type of helper plasmid might have disseminated in *Salmonella*; and (2) the helper plasmid could also undergo evolution and acquire other mobile elements, becoming a MDR plasmid (Figure 5) [15].

It appears that plasmid fusion commonly occur in bacteria, but mechanisms governing the fusion process could not be depicted prior to availability of plasmid sequencing technology [24]. Likewise, conjugative helper plasmid that facilitates the transmission of small non-conjugative IncQ types of plasmids through the formation of a conjugation pili, rather than direct fusion with the IncQ plasmid, was previously reported [25]. Fusion between conjugative plasmids had also been reported, yet our work is the first to demonstrate the existence of plasmids that play a sole functional role as a conjugative helper plasmid [26]. Sequence analysis of the sites at which a conjugative helper plasmid fuses with different ciprofloxacin resistance-encoding plasmids suggests that formation of this hybrid plasmids was due to a series of genetic events. The hot spot in the conjugative helper plasmid could be attacked by certain IS elements such as IS*26* to trigger intermolecular transposition and formation of a circular intermediate before being inserted back into the original non-conjugative plasmid through replicative transposition mechanism.

In summary, this study identified a novel type of conjugative helper plasmid that could actively fuse with non-conjugative ciprofloxacin resistance-encoding plasmids, thereby converting it into a conjugative plasmid transmissible among different species of *Enterobacteriaceae*. A more serious concern is that such fusion plasmids, which have been detectable in clinical *Salmonella* isolates since 2015, may have contributed to the dramatic increase in the incidence of ciprofloxacin resistance in *Salmonella* observable in the past few years. New strategies must be urgently devised to halt global transmission of these plasmids among *Salmonella* and other *Enterobacteriaceae* species, as well as organisms, which have already acquired such plasmids.
